# Afghanistan's Silent Epidemic: The Overuse of Antibiotics Is Fueling a Public Health Crisis

**DOI:** 10.1002/puh2.70178

**Published:** 2025-12-11

**Authors:** Mohammad Masudi, Ali Rahimi, Nasar Ahmad Shayan

**Affiliations:** ^1^ Department of Curative Medicine Faculty of Medicine Jami University Herat Afghanistan; ^2^ Department of Pediatrics Faculty of Medicine Herat University Herat Afghanistan; ^3^ Department of Epidemiology and Biostatistics Western University London Ontario Canada

Afghanistan, long plagued by conflict and economic instability, is now grappling with a less visible but equally dangerous threat: the misuse and overuse of antibiotics. The World Health Organization (WHO) warns that the rise of antimicrobial resistance (AMR) is one of the top 10 global health threats of the 21st century, and Afghanistan is a textbook case of how such problems emerge within fragile health systems [1].

Across both public and private sectors, antibiotics are prescribed at rates far beyond international recommendations. In hospitals under Kabul University of Medical Sciences, 85.5% of inpatients received antibiotics, often without confirmed diagnosis or clear justification [2]. The pattern extends to outpatient care as well. In one district hospital in Kabul, prescriptions during summer showed that 62% of patients were given antibiotics, compared to 50% in winter [3]. Another study found that drugs like ceftriaxone, metronidazole, and amoxicillin are among the most overused medications, fueling resistance [4].

Antibiotic misuse is not only a clinical issue—it is deeply cultural. Many Afghans perceive antibiotics not as targeted treatments but as general “cleaning agents” for the body, a belief documented by Médecins Sans Frontières in Ahmad Shah Baba District Hospital [5]. This mindset extends to dentistry and surgery. A randomized trial in Herat showed that prescribing antibiotics after routine tooth extraction offered no benefit, yet such prescriptions remain common [6]. Unfortunately, this mindset persists despite only moderate public awareness about antibiotic resistance. In a nationwide survey of university students, most participants had heard of the issue, but self‐medication with antibiotics was still alarmingly widespread [7].

The roots of this crisis lie in Afghanistan's weakened healthcare infrastructure. Decades of conflict have left the country with scarce diagnostic tools, weak drug regulation, and unreliable supply chains. As a result, many clinicians prescribe empirically, without laboratory confirmation, simply because no alternatives exist [1]. A hospital‐based surveillance study involving 6709 patients found that more than half of *Escherichia coli* infections were resistant to commonly used antibiotics, compared to 7%–8% in Europe. Even standard treatments like amoxicillin–clavulanic acid were largely ineffective [8]. Antibiotic misuse in Afghanistan stems from intertwined factors: weak regulation, cultural beliefs, and limited diagnostics. Over‐the‐counter sales by undertrained vendors are widespread, and public trust in antibiotics as “quick cures” reinforces demand [3]. Together, these forces sustain excessive use across hospitals, pharmacies, and communities.

Some promising tools could help curb misuse. One example is the C‐reactive protein (CRP) test, a quick finger prick used to determine whether an infection is likely bacterial or viral. A modeling study in Afghanistan estimated that using CRP tests for malaria‐negative patients could reduce unnecessary antibiotic prescriptions by about 12% [9]. While encouraging, the study noted that cost‐effectiveness depends on clinicians adhering to test results and on affordability within Afghanistan's health system. Given limited laboratory capacity [10], especially in rural clinics, nationwide use is not yet realistic. However, targeted pilot programs in better‐equipped health centers could test feasibility, training needs, and patient outcomes. If successful, these pilots could mark a practical step toward smarter antibiotic use without overpromising what current resources can deliver. Despite the scale of the problem, national responses remain fragmented. Afghanistan still lacks a comprehensive AMR surveillance system. Training in antimicrobial stewardship is limited, and public education campaigns about resistance are rare [1].

To make progress, Afghanistan needs clear, realistic actions suited to its fragile context. First, strengthen hospital‐level stewardship programs in major teaching hospitals where clinical leadership and monitoring capacity already exist [11]. Second, enforce pharmacy licensing in provincial centers and restrict antibiotic sales without prescription. Third, pilot mobile health platforms [11] that allow rural clinicians to access standard treatment guidelines and report antibiotic use patterns. Finally, Afghanistan could collaborate with WHO's Global AMR Surveillance System (GLASS) [12] and NGOs such as Médecins Sans Frontières or the French Medical Institute for Mothers and Children [13] to build reliable data systems and community awareness campaigns.

Afghanistan has faced many health crises, yet AMR presents a different kind of danger, one that grows quietly and invisibly and is easily overlooked until it becomes irreversible. Addressing it requires policy reform, public engagement, and steady investment in diagnostics and regulation. If ignored, this silent epidemic could erase decades of medical progress in Afghanistan and beyond.

## Author Contributions

Mohammad Masudi, Ali Rahimi, and Nasar Ahmad Shayan conceptualized the manuscript, wrote the original draft, supervised the process, and reviewed and edited the manuscript.

## Funding

The authors have nothing to report.

## Ethics Statement

The authors have nothing to report.

## Consent

All authors have reviewed and approved the final manuscript and consent to its publication.

## Conflicts of Interest

The authors declare no conflicts of interest.

## Data Availability

The authors have nothing to report.
